# Supplementary material for: Comprehensive Annotation and Functional Exploration of MicroRNAs in Lettuce
Source: Front Plant Sci. 2021 Dec 24;12:781836. doi: 10.3389/fpls.2021.781836 (PMC8739914; doi:10.3389/fpls.2021.781836)
Supplement: Supplementary file 3 [file Data_Sheet_9.ZIP › GSM2230751.plot/Lsa-miRN533_Lsat_1_v5_gn_7_86981.1_1447_TPlot.pdf]

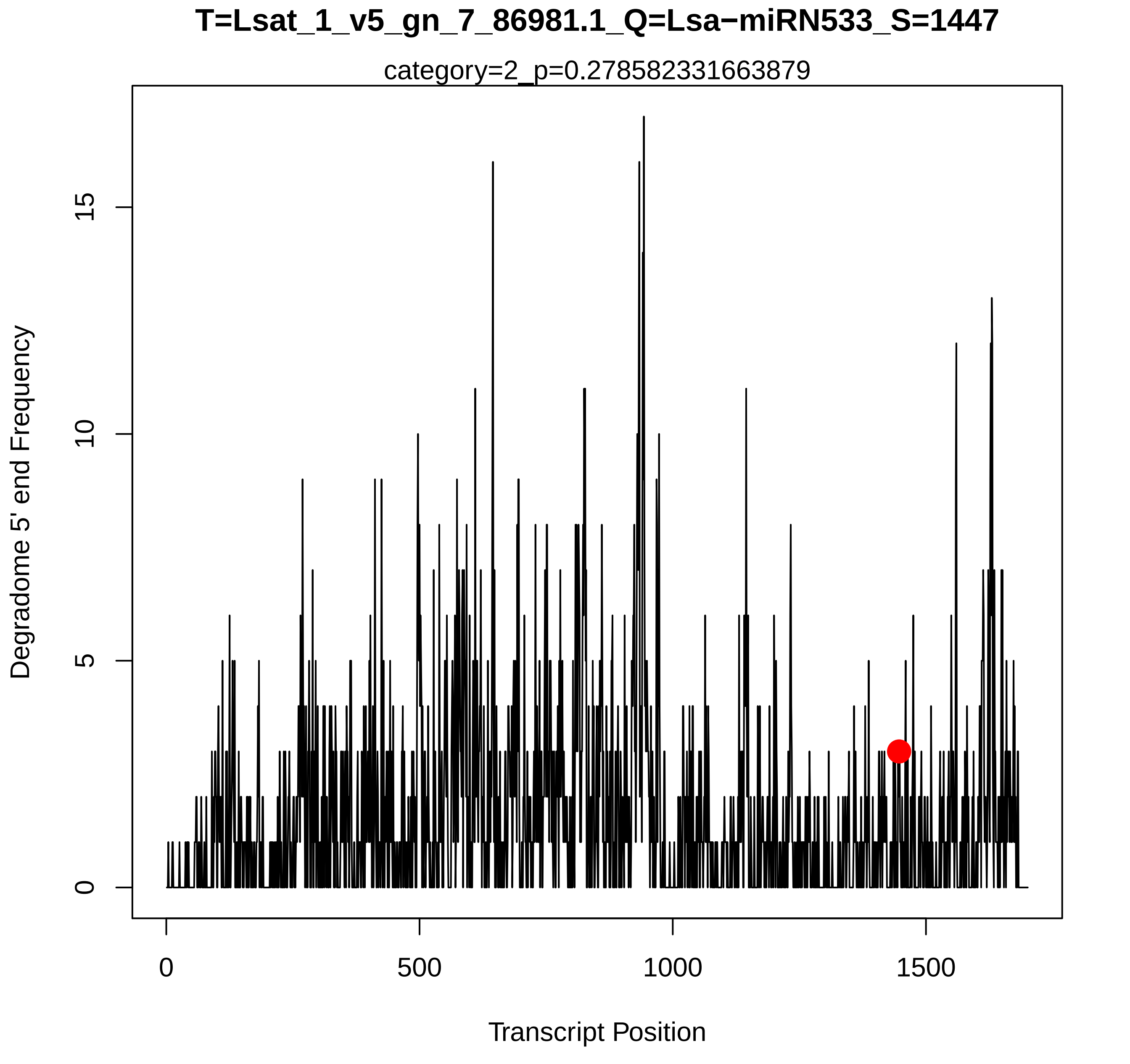
T=Lsat_1_v5_gn_7_86981.1_Q=Lsa−miRN533_S=1447
category=2_p=0.278582331663879
Degradome 5' end Frequency
0
5
10
15
0
500
1000
1500
Transcript Position
